# Photoprotective and Anti-Melanogenic Effects of Supercritical Fluids Extract from *Posidonia oceanica* Beach-Cast Leaves: From Waste Stream to Cosmeceutical Applications

**DOI:** 10.3390/md24010027

**Published:** 2026-01-08

**Authors:** Simona Manuguerra, Rosaria Arena, Eleonora Curcuraci, Concetta Maria Messina, Andrea Santulli

**Affiliations:** Laboratory of Marine Biochemistry and Ecotoxicology, Department of Earth and Marine Sciences DiSTeM, University of Palermo, Via Barlotta 4, 91100 Trapani, Italy; simona.manuguerra@unipa.it (S.M.); rosaria.arena@unipa.it (R.A.); eleonora.curcuraci@unipa.it (E.C.); andrea.santulli@unipa.it (A.S.)

**Keywords:** *Posidonia oceanica*, supercritical fluid extraction, marine bioactive metabolites, photoprotection, anti-melanogenesis, sustainable extraction

## Abstract

Marine plants are a rich source of bioactive compounds with unique properties. The Mediterranean seagrass *Posidonia oceanica* is particularly abundant in phenolics and flavonoids, which exhibit antioxidant and anti-inflammatory activities. In this study, a phenolic-rich extract (POS) was obtained from beach-cast *P. oceanica* leaves using supercritical fluid extraction (SFE), an eco-friendly technique that preserves thermolabile compounds and avoids organic solvents. POS was incorporated into a base cream (POS-enriched cream) to evaluate its bioactive potential in topical applications. The antioxidant capacity of POS and the cream formulation was firstly evaluated using the DPPH radical scavenging assay, confirming strong radical scavenging activity for the POS (IC_50_ = 2.32 ± 0.33 mg/mL) and significant activity for the POS-enriched cream (IC_50_ = 16.76 ± 0.58 mg/mL) compared to a base cream as control (IC_50_ = 37.62 ± 1.27 mg/mL). The antioxidant and photoprotective effects of POS were investigated in human skin fibroblasts (HS-68) exposed to oxidative stress and UV-induced damage, while anti-melanogenic activity was assessed in human epidermal melanocytes (HEM) by measuring tyrosinase activity and melanin content. POS significantly reduced ROS accumulation and modulated key molecular pathways involved in apoptosis (p-JNK), inflammation (NF-κB), energy balance (p-AMPK), and collagen synthesis (Col1A1) in fibroblasts. In melanocytes, both POS pure extract and POS-enriched cream effectively inhibited tyrosinase activity while maintaining unaltered basal melanin levels, indicating a modulatory rather than fully suppressive effect. These findings highlight the potential of *P. oceanica* SFE extracts as sustainable natural marine-derived products for photoprotection and anti-melanogenesis, thereby bridging the gap between marine waste stream management and applications in skin health and anti-aging strategies.

## 1. Introduction

Marine ecosystems are a promising source of bioactive compounds with distinctive chemical and biological properties that are rarely found in terrestrial environments. The diversity of marine habitats has led to unique metabolic adaptations in organisms, resulting in a wide variety of natural products with remarkable functional potential [1,2]. Marine plants and algae in particular are rich in micronutrients and secondary metabolites, such as polyphenols, flavonoids, and carotenoids, which have been associated with antioxidant, anti-inflammatory, photoprotective, and anti-wrinkle effects [3,4,5,6].

Oxidative stress and inflammation are recognized as key factors in the development of chronic diseases and dermatological disorders [7,8]. The skin, as the primary barrier against environmental insults, is particularly susceptible to oxidative injury induced by ultraviolet (UV) radiation and pollutants [9,10]. Excessive UV exposure induces reactive oxygen species (ROS) overproduction, DNA damage and activation of inflammatory cascades, accelerating photoaging and tissue degradation [8,10]. Oxidative stress is also implicated in a wide range of pathological conditions, including skin disorders and systemic diseases such as cancer, metabolic dysfunction, and cardiovascular pathologies [11,12]. Therefore, the efficacy protective strategies depends on both endogenous antioxidant defenses and exogenous supplementation provided through diet or topical formulations enriched with natural bioactive compounds [13].

Although marine plants possess significant phytochemical diversity and therapeutic potential, most research has focused on marine algae, while marine plants remain underexplored. Recent studies have identified seagrass as a promising source of secondary metabolites with potential skin-protective properties [1,3,4]. These metabolites exhibit antioxidant and anti-inflammatory activities, and have been shown to modulate molecular pathways involved in photoaging, pigmentation disorders and extracellular matrix (ECM) degradation [3,4].

Among these, *Posidonia oceanica*, a seagrass endemic to the Mediterranean, plays a key ecological role. Its meadows are among the most productive marine ecosystems, providing essential habitats and nursery grounds. They contribute significantly to oxygen production and carbon sequestration and stabilize sediments to prevent coastal erosion. Therefore, conservation of these meadows is crucial for the maintenance of regional biodiversity and the ecosystem services they provide [1,14,15,16].

In addition to its ecological role, *P. oceanica* is of significant chemical interest due to its high accumulation of secondary metabolites. These compounds include high levels of polyphenols (primarily flavonoids and their derivatives), which confer potent antioxidant properties to the biomass [1,4]. Simultaneously, substantial quantities of *P. oceanica* leaves naturally accumulate along Mediterranean coastlines. These deposits, known as banquettes, are often managed as waste, resulting in a considerable environmental and economic burden for coastal municipalities [3,4,16,17]. Management of this biomass, particularly in Italy, is further complicated by an ambiguous inconsistent legislation framework [14]. Current regulations as reflected in the legislative provisions concerning fertilizers [18,19], treat these accumulations inconsistently, oscillating between the classification of a waste product and a potential resource. Even when classified as a resource, utilization for composting is strictly limited to a maximum of 20% of the total banquette weight.

Given these challenges associated with disposal, valorizing the beach coast leaves of *P. oceanica* into high-value bioactive compounds or marine-derived natural products, used as cosmeceutical ingredients, represents a sustainable eco-friendly approach that exemplifies the principles of the circular economy, recovering the marine waste and reintegrating it into the production cycle [1,14]. Crucially, this valorization strategy is strongly supported by the confirmed therapeutic potential of the plant. Bioactive metabolites from *P. oceanica* have been reported to exert multiple protective effects on skin health. Ethanolic extracts have been shown to reduce oxidative stress and inflammation, stimulate collagen production in fibroblasts, and inhibit both tyrosinase activity and melanin synthesis, thereby exerting photoprotective and anti-melanogenic effects [3,4]. Additionally, hydrophilic extracts have been shown to inhibit the expression and activity of matrix metalloproteinases (MMP-2 and MMP-9), thereby contributing to the preservation of extracellular matrix integrity [20]. In light of the growing demand for safe and effective natural agents to counteract photoaging, recent studies have identified *P. oceanica* as a promising source of marine natural products with significant implications for skin health and well-being [3,7,16,21].

In a previous study, our research group established a sustainable protocol for collecting *P. oceanica* beach-cast leaves before full senescence, preventing their degradation and accumulation in banquettes; this approach enhanced resource valorization while providing high-quality biomass for further study [1]. We demonstrated that ethanolic extracts from beach-cast green leaves of *P. oceanica* provide high extraction yields and a remarkable polyphenol content (19.712 ± 0.496 mg GAE/g). These extracts exhibited significant biological activity, as evidenced by a DPPH radical scavenging IC_50_ value of 0.090 µg/µL. Furthermore, HPLC profiling identified chicoric acid (4991.813 µg/g) as the major phenolic compound. Preliminary investigations also showed the photoprotective capacity of these extracts in vitro in human skin fibroblasts (HS-68) exposed to UV-induced oxidative stress. However, the underlying molecular mechanisms remain poorly understood, and refined extracts obtained through sustainable processes have yet to be investigated [1].

To address this gap, we employed supercritical fluid extraction (SFE). SFE is an environmentally friendly technique that ensures high selectivity, preserves thermolabile compounds, and avoids the use of organic solvents, thereby aligning with the principles of green chemistry and strict safety standards for natural product extraction. Unlike conventional methods, SFE enables parameters such as pressure, temperature and co-solvents to be adjusted for the targeted recovery of bioactive fractions relevant to antioxidant, anti-inflammatory and photoprotective effects [22,23,24].

Building on our previous findings, this study aimed to obtain a phenolic-rich extract (POS) from *P. oceanica* leaves using SFE, and to investigate for the first time its topical application after incorporation into a base cream. The aims of this research were: (i) assessing the antioxidant and photoprotective effects of POS pure extract in human skin fibroblasts (HS-68) exposed to oxidative stress and photoaging markers; (ii) evaluating its anti-melanogenic and anti-tyrosinase activity in human epidermal melanocytes (HEM); and (iii) comparing the biological efficacy of the pure extract with that of POS-enriched cream. To reach these goals, our investigation focused on the key molecular pathways involved in oxidative stress, inflammation and anti-aging, including the expression of AMP-activated protein kinase (p-AMPK), nuclear factor-kappa B (NF-κB), phosphorylated JNK (p-JNK) and collagen 1 (Col1A1) in fibroblasts (HS-68), as well as tyrosinase activity and melanin content in melanocytes (HEM).

## 2. Results

### 2.1. Polyphenol Content and Phenolic Profile of P. oceanica Supercritical Fluid Extract

The total phenolic content and phenolic compounds of *P. oceanica* supercritical fluid pure extract (POS) are presented in Table 1.

The total phenolic content of the POS was 3.82 ± 0.43 mg GAE/g dry leaf.

Subsequent HPLC analysis revealed the presence of several individual phenolic compounds. Among the compounds identified (all expressed as µg/g of extract), ρ-hydroxybenzoic acid (396.6 µg/g) and vanillic acid (361.2 µg/g) were the most abundant, followed by chicoric (266.1 µg/g), gallic acid (249.9 µg/g) and phloroglucinol (185.5 µg/g). Moderate concentrations of ρ-coumaric (159.9 µg/g), caffeic acid (153.4 µg/g), and ferulic acid (143.8 µg/g) were also detected, while quercetin (106.1 µg/g) was present at the lowest concentration.

### 2.2. Antioxidant Activity

The antioxidant activity of POS, of control base cream and of POS-enriched cream were evaluated using the DPPH radical scavenging assay. The POS exhibited the strongest radical scavenging activity, (IC_50_ 2.32 ± 0.33 mg/mL), followed by POS-enriched cream(IC_50_ 16.76 ± 0.58 mg/m) and by control base cream, that showed the lowest activity(IC_50_ 37.62 ± 1.27 mg/mL).

### 2.3. Cytoprotective Effects in Human Fibroblasts (HS-68)

The photoprotective effect of the *P. oceanica* supercritical fluid extract (POS) was evaluated in HS-68 fibroblasts using the MTT assay. As expected, UV irradiation significantly reduced cell viability, resulting in 73% ± 5.15 viability compared to untreated controls (100% ± 0.05, *p* < 0.05).

**Figure 1:**
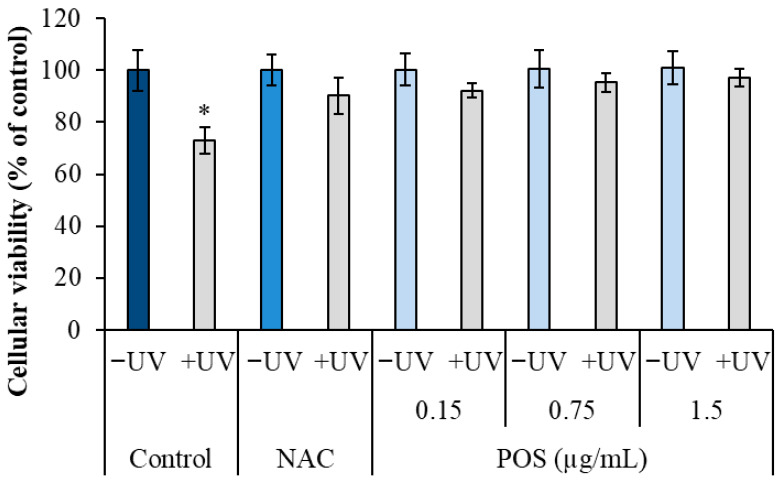
Effect of POS on cell viability in UV-irradiated HS-68 fibroblasts. Cells were pretreated with POS (0.15, 0.75, and 1.5 µg/mL) for 24 h and then exposed to UV radiation (105 erg/mm^2^/sec) for 5 min per days over three consecutive days. Cell viability was determined using the MTT assay. Data are expressed as mean ± SEM (*n* = 6). (*) Statistically significant differences (ANOVA, *p* < 0.05) compared to control.

However, pretreatment with POS significantly prior to UV exposure preserved cell viability exhibiting a clear dose-dependent protective effect (Figure 1). Notably, at the lowest concentration tested (0.15 µg/mL), POS maintained viability at 92.23% ± 2.66 compared to 73% in the UV-only group. This protective effect increased with concentration, reaching 95.23% ± 3.60 at 0.75 µg/mL and up to 97.16% ± 3.45 at the highest concentration (1.5 µg/mL). The protective effects of POS were analogous to those observed with the positive control, N-acetylcysteine (NAC), which maintained 90.23% ± 7.01 viability after UV exposure.

### 2.4. Modulation of Molecular Markers by Immunoblotting Analysis

**Figure 2:**
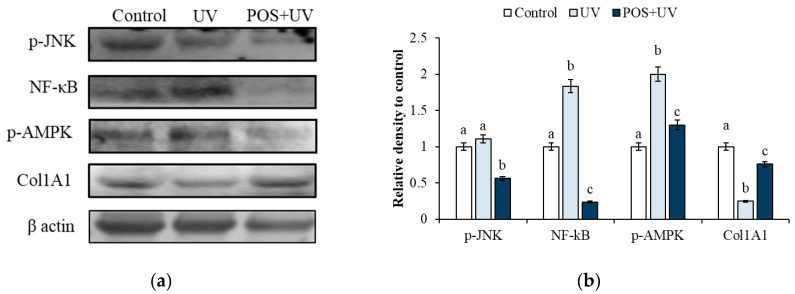
Protective effect of *P. oceanica* supercritical fluid extract (POS) against UV-induced molecular alterations in HS-68 fibroblasts. (**a**) Representative Western blot analysis of p-JNK, NF-κB, p-AMPK, and Col1A1 in control cells, UV-irradiated cells (UV), and cells pretreated with POS and subsequently exposed to UV radiation (POS + UV). (**b**) Densitometric quantification of protein expression levels. Data are presented as mean ± SD from three independent experiments. Different superscript letters indicate statistically significant differences among groups (*p* < 0.05).

To elucidate the molecular mechanisms underlying POS-mediated protection, key markers associated with oxidative stress, inflammation, apoptosis, and collagen synthesis were analyzed by Western blot. As shown in Figure 2, UV exposure significantly induced expression levels of p-JNK, NF-κB, and p-AMPK, while reducing Col1A1 protein expression compared to untreated controls (*p* < 0.05). Crucially, pretreatment with POS significantly attenuated these UV-induced alterations (*p* < 0.05) highlighting its protective mechanism.

### 2.5. Anti-Melanogenic Effect in Human Epidermal Melanocytes (HEM)

**Figure 3:**
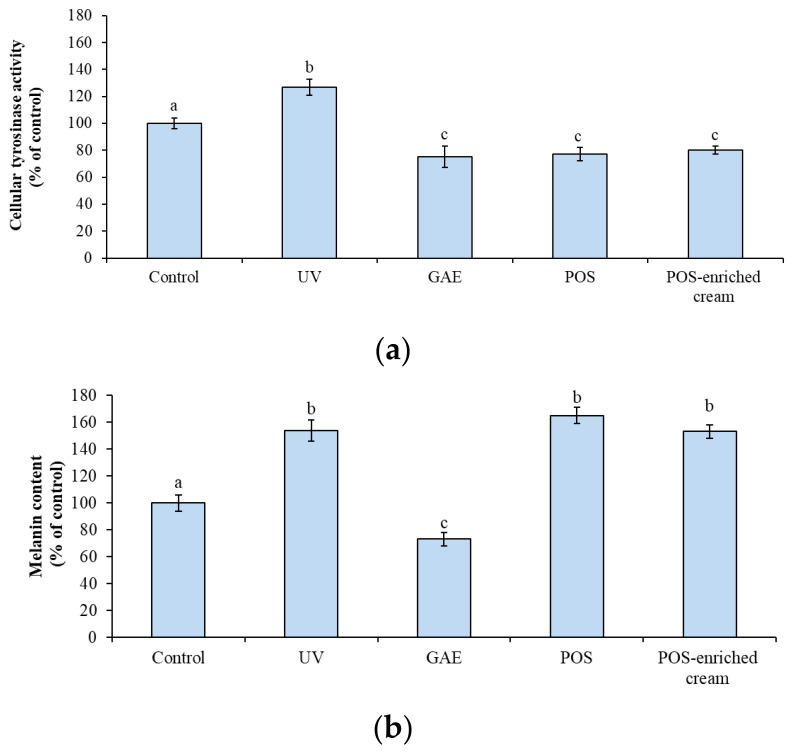
(**a**) Effect of *P. oceanica* supercritical fluid extract (POS; 0.75 µg/mL) and POS-enriched cream (100 µg/mL) on (**a**) tyrosinase activity and (**b**) melanin content in HEMs. Control: untreated cells; UV: cells exposed to UV radiation without antioxidant protection; GAE: cells pretreated with gallic acid (5 mM) and subsequently exposed to UV radiation; POS: cells pretreated with *P. oceanica* extract and UV-irradiated; POS-enriched cream: cells pretreated with POS-enriched cream and UV-irradiated. Data are expressed as percentages relative to control and represent the mean ± SEM (*n* = 6). Different superscript letters indicate statistically significant differences among groups (ANOVA; *p* < 0.05).

The anti-melanogenic activity of POS was assessed by evaluating tyrosinase activity and melanin content in HEM cells. UV exposure significantly increased tyrosinase activity by 26.78% ± 6.19 compared to control cells (*p* < 0.05). Pretreatment with either POS or POS-enriched cream significantly prevented this UV-induced activation of tyrosinase (Figure 3a). The pure extract (0.75 µg/mL) was highly effective, reducing tyrosinase activity to 77.05% ± 5.00 of the control. This level of inhibition was comparable to that achieved by the positive control, gallic acid (GAE, 5 mM), which reduced activity to 75% ± 8.14. Crucially, the POS-enriched cream (100 µg/mL) also demonstrated significant efficacy, reducing tyrosinase activity to 80% ± 3.12 of the control.

Nevertheless, treatment with POS or the POS-enriched cream did not show a significant decrease in the melanin content when compared to UV-irradiated cells (Figure 3b). While UV exposure induced a significant accumulation of melanin (to 154% of the control), the POS (165% ± 6.01) and the POS-enriched cream (153% ± 5.12) showed no reduction in the final pigment load. In contrast, the positive control, gallic acid (5 mM), significantly reduced both tyrosinase activity and melanin content to 73% ± 5.23 of the control (*p* < 0.05) (Figure 3a,b).

## 3. Discussion

It is well known that *P. oceanica* is particularly rich in antioxidant compounds that are able to counteract oxidative stress and to prevent cellular damage [4]. Ultraviolet (UV) radiation is the major source of oxidative damage in the skin, promoting the overproduction of reactive oxygen species (ROS), DNA damage, and inflammatory responses that accelerate cellular senescence and extracellular matrix (ECM) degradation [16,25,26].

This study demonstrates that *P. oceanica* extract, obtained through the eco-sustainable supercritical fluid extraction (SFE) technique, offers multifunctional protection against photoaging. The findings of this study support the use of this extract as an innovative and sustainable marine-derived ingredient, in accordance with the principles of the circular economy, by acting on both dermal structure (fibroblasts) and pigmentation mechanisms (melanocytes).

The extract produced through this environmentally friendly process exhibited a total phenolic content of 3.82 ± 0.43 mg GAE/g dry leaf (Table 1). This value is comparable to those reported in the recent literature for extracts obtained using conventional methods [27] but lower than that observed in ethanolic extracts reported in our previous study [1]. This difference can be attributed to the lower polarity of supercritical CO_2_ relative to ethanol, which leads to a more selective extraction of less polar phenolic compounds [28,29]. Similar results have been observed in other plant matrices [30,31]. Despite the lower phenolic content compared to conventional solvents, SFE offers significant advantages in terms of environmental performance and extract quality. In contrast, the use of organic solvents for extraction can raise toxicity concerns and leave residual solvents in the final product, which can lead to environmental and health issues [32]. The SFE process is solvent-free, as CO_2_ reverts to its gaseous state upon depressurization, leaving no chemical residues. This ensures a high-purity extract and aligns with green extraction principles and sustainable production practices [33,34] and, improved safety and regulatory acceptance for topical applications [35]. The phenolic profile identified in POS (Table 1) demonstrated a predominance of ρ-hydroxybenzoic acid, vanillic acid, and chicoric acid, thereby confirming that these phenolic compounds are the primary constituents of the leaves. These results are in agreement with previous literature, which reports a rich presence of phenolic derivatives and cinnamic acids in *P. oceanica*, and indicates that chicoric acid is generally the primary phenolic compound in the leaves [1,4,36].

These secondary metabolites have been demonstrated to function as pivotal elements in plant photoprotection [1,36], thus meriting consideration as prospective preventive agents against the deleterious effects of UV radiation on human skin cells.

The evaluation of the DPPH radical scavenging activity further confirms the functional potential of the POS. The IC_50_ value of 2.32 ± 0.33 mg/mL highlights a robust antioxidant activity. Although direct comparisons are limited due to the scarcity of data on extracts obtained via SFE, the results are consistent with previous findings reported for ethanol extracts of *P. oceanica* leaves. In our earlier work [1], the highest radical scavenging activity was observed in ethanol extracts from green leaves dried at 60 °C and finely ground (IC_50_ = 0.090 mg/mL), whereas the lowest activity was found in brown leaf ethanol extracts (IC_50_ = 14.530 mg/mL). The IC_50_ value of the previous ethanol extract compared to the current POS indicates that SFE is a superior method for concentrating the highly bioactive antioxidant constituents of biomass. Similarly, Kevrekidou et al. [27] reported IC_50_ values of 2850.0 ± 60.10 µg/mL and 1600 ± 75.10 µg/mL for Wet and Dry Necromass ethanol extracts, respectively. These comparisons suggest that, despite the lower polarity of supercritical CO_2_ relative to ethanol, the SFE extract maintains appreciable and indeed enhanced antioxidant capacity. The results demonstrate that SFE is a sustainable and efficient method for obtaining bioactive extracts with strong antioxidant activity. The POS retained its radical scavenging capacity within the cosmetic formulation, confirming its suitability as a functional ingredient in products.

Building upon these findings, the biological efficacy of the POS was further evaluated in human skin fibroblasts (HS-68). Pretreatment with POS conferred significant protection to cells from UV-induced damage. At the molecular level, the results of the present study demonstrate that the extract is not limited to superficial antioxidant action, but rather directly intervenes in key signaling pathways that are crucial for skin protection. Western blot analyses revealed that UV irradiation caused a significant increase in p-JNK, NF-κB, and p-AMPK, while reducing Col1A1 protein levels. Pretreatment with POS significantly attenuated these alterations, preserving cell viability and counteracting molecular signals associated with oxidative stress, inflammation, and collagen degradation.

UV-induced ROS activates mitogen-activated protein kinases (MAPKs), including JNK, which in turn regulate pro-apoptotic processes and the degradation of the extracellular matrix [37,38,39,40]. The present study demonstrates that POS significantly reduced UV-induced JNK phosphorylation, thereby limiting pro-apoptotic signaling. This finding is consistent with previous studies on polyphenol-rich natural compounds [39,40,41]. At the same time, ROS are the main activators of the transcription factor NF-κB, which is a central mediator of the inflammatory response [42]. Pretreatment with POS significantly attenuates the increase in NF-κB levels induced by UV exposure. This suggests that the bioactive compounds present in POS (such as chicoric acid, ferulic acid, gallic acid and p-coumaric acid) act upstream of these inflammatory and apoptotic cascade, blocking stress signals before they can cause widespread cellular damage [1,16,42,43]. This finding aligns with the documented anti-inflammatory properties of other natural agents, such as glycyrrhizinic acid and resveratrol, which have also been shown to inhibit NF-κB in fibroblasts and keratinocytes exposed to oxidative stress [44,45,46,47]. This multi-level mechanism makes the extract more protective than simple antioxidants, which only act as free radical scavengers. Furthermore, the study demonstrated that POS attenuated UV-induced phosphorylation of AMPK, a pivotal regulator of energy metabolism and cellular homeostasis [48]. AMPK is known to be activated under conditions of severe metabolic or oxidative stress, including UV radiation. The attenuation of this pathway by POS suggests that the extract may rapidly restore energy balance and cellular function, thereby enhancing cellular resilience and longevity against photo-damage and chronic aging processes [49]. POS also preserved the type I collagen (Col1A1), which supports the integrity and elasticity of the dermal matrix [4].

These results are consistent with previous studies that have demonstrated the capacity of natural polyphenols including epigallocatechin gallate contained in the green tea [50], anthocyanin-rich extract from a blueberry plant [39] and xanthohumol, polyphenol isolated from hops [41] to counteract UV-induced collagen degradation. Overall, these findings demonstrate that *P. oceanica* extract has multiple molecular targets and acts as a powerful cytoprotective, anti-inflammatory and anti-aging agent. The ability of the substance to modulate stress-related signaling pathways and promote collagen expression renders it a promising natural ingredient for cosmetic formulations aimed at preventing or reducing the signs of photoaging.

Building on the protective effects observed in UV-exposed fibroblasts, we next investigated the role of POS in modulating melanogenesis in epidermal melanocytes, focusing on tyrosinase activity, downstream enzymatic pathways, and melanin accumulation.

In melanocytes, POS also modulated pigmentation by influencing melanogenic enzymes. Melanogenesis is a complex physiological process primarily regulated by tyrosinase, tyrosinase-related protein 1 (TRP-1) and dopachrome tautomerase (TRP-2) [51].

Tyrosinase is involved in the first two steps of melanin biosynthesis, catalyzing the hydroxylation of tyrosine to L-DOPA and its subsequent oxidation to dopaquinone [52,53,54]. TRP-2 converts DOPAchrome into 5,6-dihydroxyindole-2-carboxylic acid (DHICA), while TRP-1 catalyzes the oxidation of DHICA to indole-5,6-quinone-2-carboxylic acid, completing the final stages of melanin synthesis [51]. Excessive activation of this pathway under UV exposure contributes to hyperpigmentation, oxidative stress, and photoaging. Consequently, tyrosinase is widely recognized as a key pharmacological and therapeutic target for skin-whitening and anti-photoaging agents [55,56].

Pretreatment with POS pure extract and POS-enriched cream significantly inhibited tyrosinase activity in UV-exposed human epidermal melanocytes (HEM), suggesting interference with the early stages of melanogenesis. A plausible mechanism involves the polyphenolic constituents of the extract, which may interact with the catalytic copper ions at the tyrosinase active site or compete with endogenous substrates, thereby reducing enzymatic activity. Such mechanisms are well-documented for marine-derived polyphenols and other natural tyrosinase inhibitors acting as copper chelators or as competitive substrates [57,58].

However, total melanin content in POS-treated cells was comparable to UV-only exposed controls. The observed dissociation between tyrosinase inhibition and melanin accumulation in POS-treated cells highlights the complex, multi-step, and redox-sensitive nature of melanogenesis. In cellular systems, inhibition of tyrosinase activity does not necessarily result in an immediate reduction in total melanin levels, which represents a cumulative and time-dependent endpoint [54]. POS may therefore primarily act as a modulator or a weak competitive inhibitor of tyrosinase, reducing catalytic efficiency without fully blocking substrate conversion. This phenomenon can be interpreted in the context of enzyme kinetics and metabolic redundancy. Phenolic compounds are well known to act as competitive or reversible tyrosinase inhibitors, interacting with the catalytic copper-containing active site or behaving as alternative substrates [59]. Accordingly, the phenolic profile of POS, which includes compounds such as chicoric and vanillic acids, supports a competitive and reversible mode of inhibition, as extensively characterized in kinetic models of other natural phenolics and glycosides [60].

In a complex cellular environment, particularly under UV-induced oxidative stress, the increased availability of endogenous substrates (e.g., L-tyrosine and L-DOPA) may partially displace such inhibitors from the enzyme active site, allowing the maintenance of basal melanin production while suppressing stress-induced enzymatic hyperactivation. Importantly, discrepancies between tyrosinase activity and melanin accumulation have been reported in cell-based kinetic studies, highlighting that enzymatic inhibition does not necessarily translate into proportional reductions in melanin content due to pathway redundancy and temporal integration of melanogenesis [61]. Consequently, although the initial and rate-limiting phase of melanogenesis is attenuated, intermediate substrates such as L-DOPA may not be completely suppressed. Under UV-induced oxidative stress conditions, characterized by elevated levels of ROS, these intermediates can undergo spontaneous oxidation or be further metabolized by downstream melanogenic enzymes, including TRP-1 and TRP-2, partially bypassing the initial enzymatic inhibition [51,62,63].

Similar discrepancies between enzymatic activity and melanin content have been reported for other natural polyphenolic modulators, supporting the concept of melanogenesis attenuation rather than complete inhibition.

In contrast, gallic acid (GAE), used as a positive control, induced a significant reduction in both tyrosinase activity and melanin content. This higher efficacy suggests a more extensive mechanism of action, presumably involving the inhibition of multiple melanogenic enzymes and/or the promotion of melanin degradation [64]. The findings of this study underscore the importance of compound potency and delivery in ensuring consistent and effective suppression of melanogenesis.

The modulatory action of *Posidonia* extract represents a safer and more physiologically compatible alternative to potent tyrosinase inhibitors such as kojic acid, arbutin and hydroquinone, which have been associated with complete depigmentation, toxicity, irritation and potential mutagenicity [54]. Since melanin constitutes the primary natural defense mechanism of the skin against damage caused by UV radiation, attenuating stress-induced hyperactivation of melanogenesis without eliminating basal melanin levels allows the extract to support a balanced state. In this state, the prevention of photoaging is achieved without compromising the skin’s intrinsic photoprotective barrier [65]. This approach appears particularly relevant given current consumer demand and regulatory trends, which increasingly prioritize biocompatible, safe, and physiologically aligned cosmetic ingredients.

Crucially, the clear efficacy of the POS-enriched cream in inhibiting tyrosinase activity confirms that the bioactive compounds extracted from Posidonia leaves maintain their functional integrity and bioavailability after its incorporation into base cream, supporting utilization of POS as a functional and stable ingredient for anti-photoaging product development.

The formulation of POS into a base cream represents a crucial factor in translating in vitro efficacy as topical application. The emulsion system acts as a protective reservoir, where excipients shield sensitive polyphenols, such as chicoric and vanillic acids, from oxidative or light-induced degradation, thereby preserving their functional integrity [66,67]. Furthermore, the matrix viscosity and the specific lipid composition play a dual role: they modulate release kinetics to ensure sustained delivery and enhance skin permeation, facilitating the diffusion of bioactives toward melanocytes in the basal epidermis and fibroblasts in the underlying dermal layers [68,69]. These factors collectively support the translational relevance of the POS-enriched cream, highlighting that its biological efficacy is a synergistic result of the extract’s potency and a stable, effective delivery system.

Overall, this study is the first to demonstrate the dual activity of the *P. oceanica* extract on complementary skin cell models, protecting the dermal structure in fibroblasts and modulating pigmentation processes in melanocytes. Its multifunctional properties highlight its potential as a bioactive ingredient suitable for formulations aimed at both photoaging prevention and the modulation of pigmentation disorders, thereby promoting skin health and homeostasis [70,71].

Future research should focus on optimizing formulation efficiency and bioavailability, and on evaluating synergistic strategies to maximize the anti-melanogenic efficacy while preserving its safety and sustainability profile.

## 4. Materials and Methods

### 4.1. Processing and Preparation of Posidonia oceanica Extract

*P. oceanica* leaves were collected from February to March 2024, along the coast of Favignana, one of the Egadi islands near Trapani (Italy), as previously described in Messina et al. [1], carefully cleaned, and freed ligules. In particular, according to the Italian legislation related to the management of *P. oceanica* banquettes, fresh foliar bundles naturally detached from the meadow (e.g., due to sea storms) and not yet deposited along the coast, were collected, before forming.

In laboratory, the green leaves were dried in a ventilated oven (Termaks TS 800, Bergen, Norway) at 37 °C until a constant weight was achieved, then ground using a Grindomix homogenizer (Retsch GM 200, Haan, Germany) (7000 rpm, 2 min). The resulting powder was fractionated using a sieve column (Retsch AS 200 Control, Haan, Germany) and 250–500 µm particle size fraction was selected for Supercritical Fluid Extraction (SFE), following a standardized procedure [23,30,31,72].

#### Supercritical Fluid Extraction (SFE)

SFE was performed using a Helix System Basic Model (Applied Separation, Allentown, PA, USA). Supercritical CO_2_ was used as the extraction solvent without any organic modifiers or co-solvents. For each extraction 40 g of leaf powder was mixed with an equal volume of diatomaceous earth, in a ratio 1:1 (*v*/*v*). The extraction process was conducted in two consecutive phases: a static phase (1 h at 50 °C and 280 bar) to allow the initial compound solubilization, followed by a dynamic phase (2 h) with a continuous flow of supercritical CO_2_ (4 L/min). During this phase, the extract vessel was maintained at 50 °C and 280 bar, while the separator vessel was maintained 50 °C and 150 bar to precipitate and collect the extract. The extract was collected in the separator vessel and stored at −20 °C [73].

### 4.2. Total Polyphenols Contents

The total phenolic content (TPC) of the samples was determined using the Folin–Ciocalteu colorimetric assay [74,75]. Gallic acid (Merck KGaA, Darmstadt, Germany) was used as the calibration standard at concentrations ranging from 5 to 500 mg/mL. The results were expressed as milligrams of gallic acid equivalents (GAE) per gram of *P. oceanica* dry weight (dw) [2,31,76,77]. The absorbance of each reaction mixture was measured at 725 nm using a Multiskan Sky Microplate Reader (Thermo Scientific™, Waltham, MA, USA). All measurements were performed in triplicate.

### 4.3. High-Performance Liquid Chromatography (HPLC) Analysis

The phenolic profile of POS was determined according to analytical procedures previously described [1]. The extract was standardized based on its phenolic profile to ensure reproducibility across different extraction samples. The POS solution (10 mg/mL) was filtered through a 0.2 µm syringe filter (MiniUniprep™, Whatman, Maidstone, UK) prior to injection. The analyses were performed using an Elite LaChrom RP-HPLC system (Merck–Hitachi, Tokyo, Japan) equipped with an L-7100 pump, L-7350 column oven, and L-2455 diode array detector (DAD). Data acquisition and processing were conducted utilizing EZChrom Elite software (version 3.1.7, Scientific Software, Pleasanton, CA, USA). The chromatographic separation was achieved on a Kinetex C18 column (150 × 4.6 mm, 5 µm particle size, 100 Å pore size; Phenomenex, Torrance, CA, USA) maintained at 35 °C. The mobile phase consisted of solvent A (0.1% aqueous H_3_PO_4_, Merck, HPLC grade) and solvent B (methanol, Chromanorm, VWR Chemicals, HPLC grade). The binary gradient, was programmed as follows: 0–3 min: 5% B; 3–6 min: 5–25% B; 6–9 min: 25–37% B; 9–13 min: 37% B; 13–18 min: 37–54% B; 18–22 min: 54% B; 22–26 min: 54–95% B; 26–29 min: 95% B; 29–29.15 min: 95–5% B; 29.15–36 min: 5% B. The flow rate was 1.5 mL/min and the injection volume was 20 µL.

Detection was performed at 205, 280, 320 and 350 nm. Phenolic compounds were identified and quantified by comparison with authentic HPLC-grade standards (Merck KGaA, Darmstadt, Germany), including Caffeic Acid, ρ-coumaric Acid, Gallic Acid, ρ-hydroxybenzoic Acid, Phloroglucinol, Quercetin, Trans-ferulic Acid and Vanillic Acid, according to the procedure described by Robbins and Bean [78]. Calibration curves were established using standard solutions of methanol at concentrations ranging from 0.0125 to 0.5 mg/mL. All samples were analyzed in triplicate, and each injection was performed in duplicate to evaluate both analytical repeatability and extraction reproducibility. Results are reported as mean ± standard deviation (SD).

### 4.4. DPPH Assay

The DPPH (1,1-diphenyl-2-picrylhydrazyl) radical scavenging assay was performed as previously described by Messina et al. [1], with minor modifications according to Bernatoniene et al. [79]. Briefly, 400 µL of different concentrations of POS pure extract (0.5–10 mg/mL) and base cream control and POS-enriched cream (containing 1% POS) at 1–10 mg/mL were mixed with 1.6 mL of 0.1 mM DPPH solution (Merck KGaA, Darmstadt, Germany) in ethanol and incubated for 30 min in the dark. A blank containing the sample at the corresponding concentration in ethanol was prepared under the same conditions to eliminate possible sample color interference [80].

The absorbance was read against the blank at 517 nm using a microplate reader (Multiscan-Sky Microplate Reader, Thermo-Scientific™, Waltham, MA, USA). Gallic acid was used as the reference standard. The following Formula (1) was used to calculate the percentage of inhibition (I%) of free radicals DPPH:

(1) $$I\%=1-\left(\frac{A\;sample}{A\;blank}\right)\times 100$$

where A blank is the absorbance of the control reaction, and A sample is the absorbance of the test sample. The inhibition percentage was calculated for all concentrations (1 to 10 mg/mL) used. The IC_50_%, which is the concentration when 50% of the antioxidant is reduced was calculated as described by Yeddes et al. [81].

### 4.5. Cell Lines and Culture

Human skin fibroblast (HS-68) cells (ECACC No. 89051701, Sigma-Aldrich, Saint Louis, MO, USA) were cultivated in Dulbecco’s Modified Eagle’s Medium (DMEM; Merck, Darmstadt, Germany) supplemented with 10% fetal bovine serum (FBS), 2 mM L-glutamine, and 100 µg/mL penicillin–streptomycin.

Human epidermal melanocytes (HEM) from adult donors (catalog No. 104-05A, Cell Applications, Inc., San Diego, CA, USA) were maintained in Melanocyte Growth Medium (MGM; Merck, Darmstadt, Germany). The cells were cultivated at a temperature of 37 °C in a humidified incubator with 5% CO_2_; the medium was replaced every 2–3 days.

### 4.6. Preparation of Base Cream Control and POS-Enriched Cream for In Vitro Assays

POS-enriched cream was prepared dispersing POS pure extract in a base cream at a final concentration of 1.0%.

For in vitro cell treatments, emulsions of base cream control and POS-enriched cream were prepared following the methodology of Zanatta et al. [52]. Briefly, each cream was dispersed in sterile phosphate-buffered saline (PBS) to make a homogeneous emulsion stock. This stock was then solubilized in dimethyl sulfoxide (DMSO) to a final concentration of 0.1% (*v*/*v*) and subsequently diluted in MGM medium to achieve a final test concentration of 100 µg/mL of cream for cell assays.

### 4.7. HS-68 Fibroblast Treatment and UV Irradiation

HS-68 fibroblasts were seeded at a density of 8000 cells/well in 96-well plates and incubated overnight. Subsequently, cells were pretreated with increasing concentrations of POS (0.15, 0.75 and 1.5 µg/mL) for 24 h prior to UV-irradiation. N-acetylcysteine (NAC 5 mM), a known synthetic antioxidant, was used as a positive control.

The UV irradiation protocol simulated photoaging via daily exposure for three consecutive days. Before each daily irradiation, the culture medium was replaced with Phosphate-Buffered Saline (PBS). Cells were irradiated using a KW 254 nm UV lamp at a dose rate of 105 erg/mm^2^/s. The exposure time was progressively increased: 5 min on Day 1, 10 min on Day 2, and 15 min on Day 3 [1,25,82]. Non-irradiated cells and untreated cells were included as controls. Cell viability was assessed using the MTT assay [83] and expressed as a percentage relative to the untreated control group.

#### Western Blot Analysis

HS-68 fibroblasts were seeded in 25 cm^2^ flasks and treated with POS (0.75 µg/mL) for 24 h, followed by UV irradiation as previously described. Proteins were separated by SDS-PAGE on precast gels (Bio-Rad, Hercules, CA, USA) and transferred onto nitrocellulose membranes using a Trans-Blot Turbo Transfer System (Bio-Rad). Protein loading was verified by Ponceau S staining (Merck, Darmstadt, Germany). Membranes were incubated with primary antibodies specific for AMPK, NF-κB (rabbit polyclonal), p-JNK, Col1A1 and β-actin (mouse monoclonal) (Merck, Darmstadt, Germany) at the dilutions recommended by the manufacturers. Appropriate horseradish peroxidase-conjugated (anti-mouse or anti-rabbit, GAR/M-HRP, Bio-Rad, Hercules, CA, USA) or alkaline phosphatase-conjugated secondary antibodies were applied according to the origin of the primary antibody. Protein signals were detected using enhanced chemiluminescence (ECL) reagents (Clarity Western ECL Blotting Substrate, Bio-Rad, Hercules, CA, USA) and BCIP/NBT substrate system (Bio-Rad, Hercules, CA, USA). The images were acquired using the Chemi Doc XRS system (Bio-Rad, Hercules, CA, USA) and analyzed with Image Lab software (Bio-Rad, Hercules, CA, USA). All experiments were performed in triplicate. Protein expression levels were quantified as fold changes relative to control, and results are expressed as mean ± SD of three independent immunoblots.

### 4.8. HEM Cell Treatment and UV Irradiation

HEM cells were seeded at a density of 1.5 × 10^5^ cells/flask in 25 cm^2^ flasks and incubated for 24 h. Cells were subsequently pretreated with POS (0.75 µg/mL) or POS-enriched cream (100 µg/mL) prior UV irradiation.

UV irradiation was performed daily for three consecutive days using a 254 nm KW lamp at a dose of 105 erg/mm^2^/sec for 30 min [1,52]. Gallic acid (GAE, 5 mM) was included as positive control. Following irradiation, cells were incubated overnight at 37 °C before collection [51].

The anti-melanogenesis activity was assessed via cellular tyrosinase activity and melanin quantification assay.

#### 4.8.1. Cellular Tyrosinase Activity Assay

Tyrosinase activity in HEMs was determined spectrophotometrically by monitoring the oxidation of L-DOPA (3,4-dihydroxy-L-phenylalanine) to DOPA-chrome. After treatment, cells were washed with sterile PBS and lysed in 100 mM sodium phosphate buffer (pH 6.8) containing 1% Triton X-100 and 0.1 mM PMSF. Lysates were centrifuged at 13,000 rpm for 20 min at 4 °C.

For the enzymatic assay, an aliquot of 100 µL of lysate (containing 100 µg of total protein) was incubated with 100 µL of 5 mM L-DOPA (Merck, Darmstadt, Germany) in 96-well plates. The reaction mixture was incubated at 37 °C for 2 h.

The resulting DOPA-chrome formation was monitored by measuring the absorbance at 475 nm using a Multiskan Sky Microplate Reader (Thermo Scientific, Waltham, MA, USA) [51].

#### 4.8.2. Measurement of Melanin Content

After the treatment, HEM cells were detached using trypsin/EDTA solution, washed twice with sterile PBS, and pelleted by centrifugation. Melanin was extracted by resuspending the pellets in 2 M NaOH and incubating at 100 °C for 30 min. The resulting extract was transferred to a microplate, and the absorbance was measured at 405 nm using a Multiskan Sky Microplate Reader (Thermo Scientific™, Waltham, MA, USA). Intracellular melanin content was quantified by comparison with a standard curve generated using synthetic melanin (Sigma-Aldrich, Dorset, UK) [51].

### 4.9. Statistical Analysis

Data are expressed as mean ± standard deviation (SD) in tables, while figures report mean ± standard error of the mean (SEM). Statistical differences were evaluated for each parameter using one-way analysis of variance (ANOVA). Post hoc comparisons among mean values were performed using either the Student–Newman–Keuls test or the Games–Howell test, depending on the results of the homogeneity of variance, which was verified by Levene test. The significance level of *p* < 0.05 was considered statistically significant. All statistical analyses were performed using SPSS software for Windows^®^ (version 20.0; SPSS Inc., Chicago, IL, USA).

## 5. Conclusions

Our study demonstrated that the supercritical fluid extract of *Posidonia oceanica* (POS) exhibits multiple protective effects against UV-induced skin damage, validating a sustainable, non-toxic extraction methodology. In human fibroblasts, POS key pro-inflammatory and stress signaling pathways, specifically NF-kB and p-JNK, while contributing to preservation of collagen integrity (Col1A1). Notably, both POS pure extract and POS-enriched cream inhibited tyrosinase activity in melanocytes. These results represent the first evidence that *P. oceanica* not only safeguards dermal structure but also safely modulates the enzymatic initiation of melanogenesis. This dual capacity indicates POS as an extremely promising marine-derived natural product for the development of innovative topical cream formulations. The success of this valorization effort reinforces the potential of marine biorefineries and underscores the critical need to develop sustainable, marine-based products for skin health. Our work emphasizes how the continued study of marine biodiversity and its sustainable management can be directly and profitably linked to the creation of advanced photoprotection and anti-aging strategies, effectively transforming a waste biomass into a high-value resource.

## Figures and Tables

**Table 1:** Polyphenol content (mg GAE/g *P. oceanica* leaf) and concentrations (µg/g) of standard phenolic compounds in *P. oceanica* leaf extracts obtained by SFE.

Sample	Total Phenolic Content (mg GAE/g)	Phenolic Compounds	Concentration (µg/g)
POS	3.82 ± 0.43	Phloroglucinol	185.5 ± 19.97
		Gallic acid	249.9 ± 29.69
		ρ-hydroxybenzoic acid	396.6 ± 22.45
		Vanillic acid	361.2 ± 48.69
		Caffeic acid	153.4 ± 9.97
		ρ-coumaric acid	159.9 ± 12.05
		Ferulic acid	143.8 ± 9.39
		Chicoric acid	266.1 ± 30.91
		Quercetin	106.1 ± 13.64

Date are reported as means ± SD.

## Data Availability

The original contributions presented in this study are included in the article. Further inquiries can be directed to the corresponding author.

## Abbreviations

The following abbreviations are used in this manuscript:

SFE	Supercritical Fluid Extraction
DPPH	1,1-diphenyl-2-picrylhydrazyl
POS	SFE *Posidonia oceanica* extract
HS-68	Huma skin fibroblast
HEM	Human epidermal melanocyte
AMPK	AMP-activated protein kinase
NF-κB	nuclear factor-kappa B
p-JNK	phosphorylated JNK
COl1A1	collagen 1
